# Assessing pharmaceutical consultations: Comparing pharmacy-recommended medications for minor ailments and regulatory compliance in a Latin American healthcare network

**DOI:** 10.1016/j.rcsop.2023.100300

**Published:** 2023-07-06

**Authors:** Esteban Zavaleta-Monestel, Jorge Arturo Villalobos-Madriz, Bruno Serrano-Arias, Sebastián Arguedas-Chacón, José Pablo Diaz-Madriz, Mery Alejandra Ferreto-Meza, Betzy María Romero-Chavarría, Priscila Zumbado-Amerling

**Affiliations:** a Department of Pharmacy, Hospital Clinica Biblica, San José, Costa Rica; b Faculty of Pharmacy, Universidad de Ciencias Médicas, San José, Costa Rica

**Keywords:** Community pharmaceutical services, Pharmacist, Primary health care, Pharmaceutical care, Costa Rica

## Abstract

**Background:**

The importance of access to medicines in promoting global health cannot be overstated, particularly as an estimated 2 billion people lack access to basic medicines, particularly in developing nations. While over-the-counter (OTC) medications are relatively safe and cost-effective, there is a risk of misuse due to factors such as inaccurate self-diagnosis, inadequate dosing, addiction, adverse drug reactions, and drug interactions. To ensure proper use and prevent irrational self-medication, pharmacists can play a crucial role in guiding patients. However, the legislation in Costa Rica only covers OTC and prescription drugs, and health authorities are proposing a new decree to include a list of drugs that can be recommended by pharmacists without a prescription to treat minor ailments, which would be classified as behind-the-counter (BTC).

**Objective:**

Characterize the pharmaceutical consultation, compare the medications recommended by pharmacy professionals for minor ailments with the legislation in force in Costa Rica, and determine whether the current OTC medications are sufficient to treat minor ailments.

**Material and methods:**

This study is a descriptive, observational, cross-sectional study that focuses on a sample of the Costa Rican population that comes to consult with a pharmacist in one of the four pharmacies of the Hospital Clínica Bíblica (HCB) in San José, Costa Rica. Consultations included users over 18 years of age or caregivers of underage patients seeking advice or assistance from publicly accessible pharmacies, excluding consultations that involved information related to other hospital departments. This study aims to analyze the pharmaceutical consultation for minor ailments and compare the medications recommended by pharmacists with the list of medications allowed in Costa Rica. The study also aims to determine if the current OTC medications are adequate for treating minor ailments.

**Results:**

A total of 1537 consultations were gathered, which were divided into four categories: pharmaceutical recommendation (48%), medication information (31%), other consultations (18%), and referrals to another health professional (3%). Among the consultations classified as pharmaceutical recommendations, 90% were related to minor ailments. Prescription drugs accounted for 75.3% of the medications recommended and consulted. However, when the BTC category was included, the percentage of recommended prescription drugs decreased to 29.6%, while BTC drugs constituted 45.7%. Finally, the chi-square test rejected the null hypothesis that there was no association between the availability of OTC drugs and the minor ailments for which patients sought consultation.

**Conclusions:**

Most cases of pharmacy consultations involve minor illnesses such as digestive symptoms, joint pain, and respiratory issues. The proposed decree by health authorities in Costa Rica is noteworthy as it establishes standardized protocols for the prescription of BTC medications to ensure the safety of patients.

## Highlights

• Pharmacy consultations focus on minor symptoms; common in 18–65 age group, 75.3% of drugs advised need a prescription.
• When considering the proposed BTC category, the percentage of prescription drugs decreased to 29.6%, while BTC drugs constituted 45.7%.

## 1 Introduction

Access to medicines is fundamental for any health system worldwide because it is an indicator of the health of the population due to its role in promoting health maintenance.^1^^,^^2^ Approximately 2 billion people lack basic medicines and affordable and quality medicines are limited, especially in developing countries.^3^

Prescription medications are drugs that can only be obtained with a prescription from a healthcare professional, usually used to treat complex or serious medical conditions. They require professional evaluation and guidance due to their potentially harmful side effects. On the other hand, over-the-counter (OTC) medications are available for purchase without a prescription and are intended for self-diagnosis and treatment of common ailments such as headaches, allergies, and minor pain. OTC medications have lower doses of active ingredients and come with clear instructions and warnings for safe use by consumers. It is crucial to follow the guidance of healthcare professionals and adhere to the instructions on OTC medication labels to ensure appropriate and safe usage.^4^^,^^5^

Conversely, since over-the-counter medications are available to the public, there is a potential for misuse. The absence of a requirement for a medical consultation may lead to inaccurate self-diagnosis, masking of underlying conditions, incorrect dosing, addiction problems, adverse drug reactions, and drug interactions.^4^^,^^6^^,^^7^ A study conducted in Latin America revealed that 78.4% of patients suffering from chronic pain engage in self-medication using analgesics.^8^

Pharmacists can help prevent problems arising from uninformed self-medication, ensuring the appropriate use of medications.^9^ Countries such as the United Kingdom, France, and Canada, have opted for the creation of a third category, called behind-the-counter (BTC) medicines. Drugs within this category, like OTCs, do not need a prescription for dispensing but require professional supervision to be dispensed.^4^^,^^5^^,^^10^

In this context, pharmacists are vital for ensuring the appropriate use of medications, preventing problems arising from uninformed self-medication, and providing professional supervision during the dispensing process due to their extensive knowledge and expertise in medications, interactions, and their appropriate use. While pharmacy assistants and technicians play important supporting roles, it is the pharmacists who possess the expertise to make informed decisions and provide optimal pharmaceutical care for patients.^5^^,^^10^

Latin America encounters numerous hurdles when it comes to healthcare accessibility, which can be attributed, at least in part, to socioeconomic factors.^1^^,^^11^, ^12^, ^13^ In particular, approximately 25% of the population residing in rural and remote areas confronts insufficiencies in accessing essential healthcare services.^14^ The region contends with an array of challenges, including socioeconomic disparities that affect health outcomes, discrepancies in the quality of healthcare provisions between the public and private sectors, and inequitable financing mechanisms, among other pressing concerns.^13^ In addition, compared to other regions, there are more medicines available without a prescription in Latin American countries, which encourages patients to seek advice on medications and illnesses from pharmacists in communal pharmacies. These pharmacies serve large segments of the population, particularly poor and uninsured patients.^1^^,^^11^^,^^12^ In Costa Rica, BTC medicines can be found not only in pharmacies but also in supermarkets, convenience stores, gas stations, and mini-marts.

Pharmacy consultations have become a readily available and immediate service, making it the primary place where patients seek early resolution of their health issues. Health professionals are vital in this context since the misuse of OTC drugs leads to over 170,000 hospitalizations per year in the US, costing USD 750 million annually.^15^

Pharmacists and trained healthcare professionals offering medication referral services have been found to enhance medication adherence, treatment satisfaction, and overall health outcomes, resulting in reduced healthcare costs.^5^^,^^6^ While these services facilitate prompt access to medications for the general population, it's important to note that not everyone has immediate access to healthcare facilities for addressing minor ailments which are defined as mild or less severe manifestations of a condition or illness. These symptoms may not significantly impact the overall health or daily functioning of an individual and often do not require immediate medical intervention. In such cases, pharmacies play a crucial role in providing essential support.^16^^,^^17^

The legislation in Costa Rica only contemplates OTC and prescription drugs. No category includes medicines indicated after a pharmaceutical consultation or BTC medicines like in other countries. However, health authorities are preparing a decree to establish a list of pharmacist-recommended medicines that do not require a prescription. Additionally, in the country the Ministry of Health authorizes the acquisition of Levonorgestrel in pharmacies without a prescription, making it the medication under pharmaceutical recommendation.^18^

This study aims to analyze the pharmaceutical consultation for minor ailments and compare the medications recommended by pharmacists with the list of medications allowed in Costa Rica. The study also aims to determine if the current OTC medications are adequate for treating minor ailments.

## 2 Materials and methods

The current work contemplates a descriptive, observational, cross-sectional study which is constituted by a sample of the Costa Rican population that consults with a pharmacist in one of the pharmacies of the Hospital Clínica Biblica (HCB) a private hospital open to the general public, which has 4 pharmacies. The research adhered to the guidelines known as Strengthening the Reporting of Observational Studies in Epidemiology (STROBE).^19^

### 2.1 Inclusion criteria

a. Users over 18 years of age or caregivers of underage patients who consult the pharmacist.
b. Seek advice or assistance from one of the publicly accessible pharmacies at Hospital Clínica Bíblica.
c. Consult one of the pharmacies of a private hospital in Costa Rica open to the public:

• Central Pharmacy: Monday to Saturday from 9 am to 7 pm.
• Omega Pharmacy: Monday to Friday from 9 am to 7 pm.
• San José Emergency Pharmacy: Open 24 h every day of the week.
• Santa Ana Emergency Pharmacy: Open 24 h every day of the week.

d. Consultations (including every question regarding any medication, or formulation sold in pharmacies) made to the pharmacist in person or by telephone of pharmacotherapeutic, non-pharmacotherapeutic, or administrative type.

### 2.2 Exclusion criteria

a. Consultations with pharmacists that include information related to other departments of the hospital (laboratory, emergency, admission, nursing, medical direction).
b. Queries that were inadequately registered in the data collection tool, lacking the necessary information in the required spaces.

### 2.3 Data collection and statistical analysis

The data for the study was collected by pharmacists at the Hospital Clínica Bíblica. They entered the data into a digital form designed for this purpose. The form was filled out solely by the attending pharmacist, and the collected information was anonymous and only accessible to the research group digitally. A consecutive number was assigned to each recorded consultation entry.

During the pharmacy consultation, the following information was collected, including the pharmacist ID, start time, and end time of the consultation. Additionally, the sex and age range of the patient were recorded. The type of query was also noted, with options including pharmacotherapeutic consultation, non-pharmacotherapeutic consultation, and administrative consultation.

The symptoms for which the patient is consulting, and the duration of the issue were recorded, along with any medication recommended by the pharmacist, whether OTC or with a medical prescription, according to the current national law. It is also crucial to document any allergies the patient may have to medicines, as well as any other conditions they may be experiencing. If the patient were pregnant or breastfeeding, this must be specified as well.

In some cases, it was necessary to refer the patient to another healthcare service seeking medical evaluation due to the characteristics of their symptoms. When making a referral, it was important to clearly state the criteria by which the patient was being referred to another health professional.

The sample size was determined by all pharmaceutical queries or recommendations registered on the Microsoft Forms platform that met the inclusion and exclusion criteria.

After collecting the data, it was processed to standardize the entries and add the ATC classification.^20^ Then this, information was displayed in a visual representation using charts or graphs to obtain information on the main types and symptoms of pharmaceutical consultations, the medicines advised in consultations by pharmacists, and medicines asked for by patients. The percentage of indicated medicines that corresponded to the legislation for OTC or prescription sale was compared, and the data was analyzed to determine what percentage of active ingredients were within the proposals for the creation of the BTC classification, along with the protocols of pharmaceutical indication.

The proposal of the BTC list compared includes groups of drugs such as NSAIDs, H1 antihistamines (first and second generation), cough suppressants, contraceptives, anti-migraines, pump inhibitors, antiemetics, H2 blockers, anti-ulcers, laxatives, anti-hemorrhoids, vaccines, lubricants for dry eyes, antivirals against cold sores, immunostimulants, probiotics, anti-varicose drugs, and drugs for irritable bowel syndrome. Additionally includes topical medications for scarring, vaginal fungal infections, acne, and burns.

A chi-square analysis was conducted to test the hypotheses related to the current Costa Rican OTC drug list. The null hypothesis (H0) proposed that the availability of drugs that are legally registered as OTC and the minor ailments for which the patient consults are independent, while the alternative hypothesis (H1) proposed that the availability of OTC drugs and the minor ailments for which the patient consults are not independent. If H0 is rejected, it would indicate that the variables are associated, which can be interpreted as that the current OTC drugs are related to the queries due to minor ailments in Costa Rica.

### 2.4 Ethical considerations

This study was approved in session CI-006-2022 by the Scientific Ethical Committee of the University of Medical Sciences (CEC-UCIMED). Written consent was not necessary for this study.

## 3 Results

### 3.1 Analysis of consultations and symptom distribution in the dataset

The total number of entries collected was 1537 within they were grouped into four categories: pharmaceutical recommendation (48%), medication information (31%), other consultations (18%), and referrals to another health professional (3%).

**Fig. 1:**
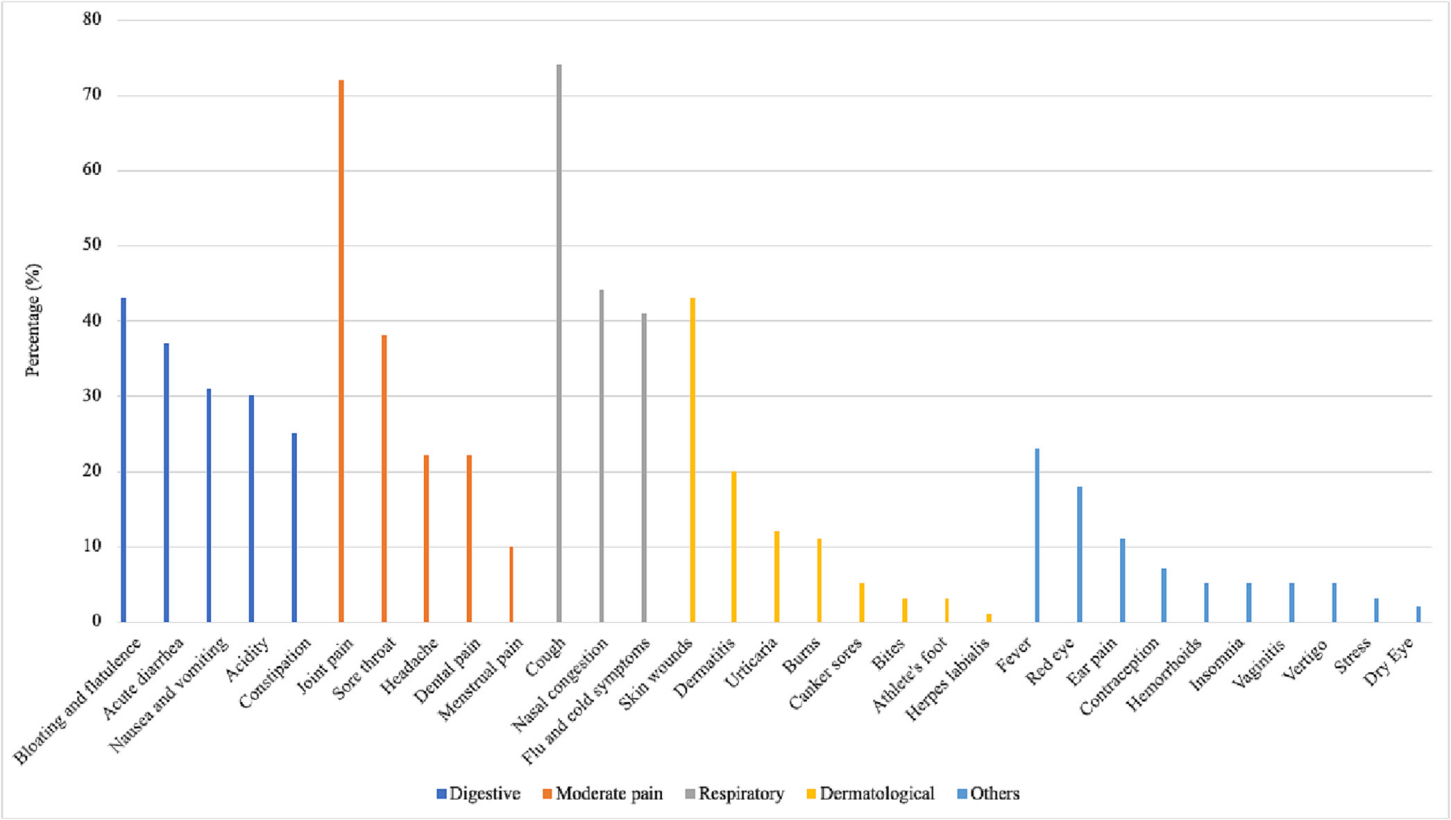
Main types and symptoms of pharmaceutical consultations.

Fig. 1 presents the main types of consultations and their corresponding symptoms. The data shows that the symptom with the highest number of consultations was distension and flatulence in the digestive category, accounting for 26% of all consultations. In the moderate pain category, joint pain was the most consulted symptom, with 44% of all consultations. Cough was the most frequently consulted symptom in the respiratory category, accounting for 47% of all consultations. In the dermatological category, consultations for skin wounds were the most frequent, with 44% of all consultations. Lastly, fever was the most consulted symptom in the “other” category, accounting for 27% of all consultations.

Out of a total of 743 consultations that involved pharmaceutical recommendations, 90% were classified as minor ailments. These minor ailments were further grouped into five categories, namely digestive (25%), moderate pain (24%), respiratory (24%), dermatological (15%), and others (12%). Many consultations (73%) fell into the categories of digestive, respiratory, and moderate pain.

In terms of the mode of sale, 75.3% of both recommended and consulted medicines were prescription drugs, while 24.7% were available over the counter. Upon a second analysis, considering the proposal of the health authorities of Costa Rica to include the BTC category, the percentage of recommended drugs that require a prescription decreased to 29.6%, while BTC drugs constituted 45.7%.

### 3.2 Analysis of association between OTC drug availability and type of minor ailments

When applying the chi-square test, a significant relationship between the type of symptoms and whether the indicated drugs were available (OTC or not) was discovered. This finding challenges the assumption that these variables are independent. The statistical analysis, specifically the chi-square test, revealed a *p*-value well below the predetermined significance level (0.05), providing strong evidence against the null hypothesis. In other words, there is a clear association between the availability of OTC drugs and the type of minor ailments.

### 3.3 Analysis of medication information consultations by age range and ATC 1st level classification

**Fig. 2:**
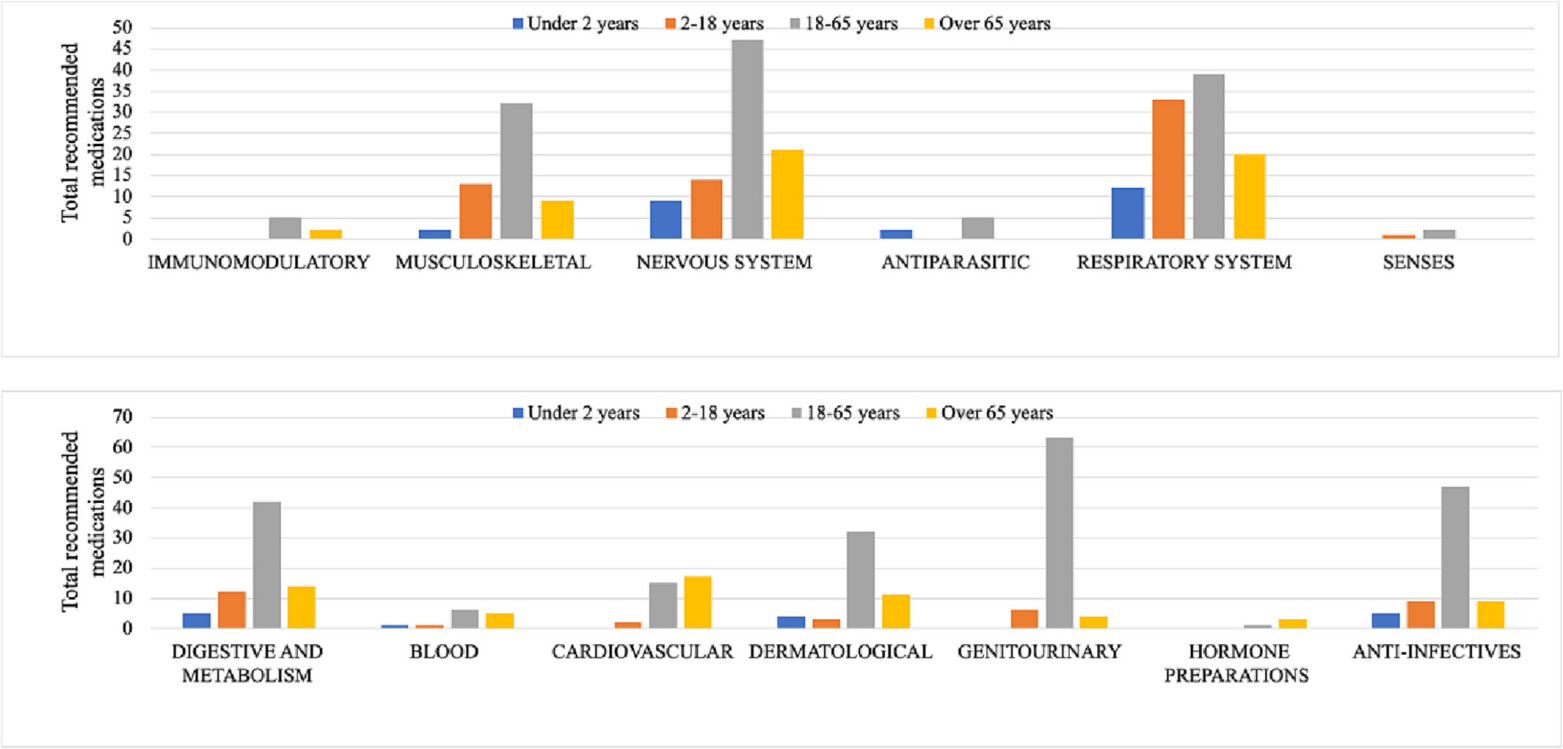
Relationship between age range and recommended ATC 1st level classification.

A total of 594 consultations were made for medication information. Fig. 2 displays the consultations categorized by age range and ATC 1st levelclassification, as consulted by patients. The age group with the highest concentration of consultations was 18–65 years (58%), followed by over 65 years (18%), then 2–18 years (15%), and finally, consultations for children under 2 years (7%). Among the population over 65 years of age, the most consulted category was the Nervous System with 18%, followed by the Respiratory System with 17%. For the 18–65 age range, the most notable categories were: genitourinary system and sex hormones with 18%, Nervous System (14%), and Anti-infectives for systemic use with 14%. In the 2–18 years range, the most consulted category was the Respiratory System (35%), and finally, in children under 2 years, the primary category was the Respiratory System (30%).

### 3.4 Breakdown of medication information consultations by inquiry type

As presented in Table 1, out of the total 594 drug consultations for information, 29% was solely related to dosage inquiries. Meanwhile, 28% of consultations were for general information, which may include multiple queries about the same medication such as dose, usage, frequency, adverse reactions, interactions, contraindications, and pharmaceutical alternatives. Additionally, 15% of consultations concerned both medication usage and dosage.

**Table 1:** Information on the consulted medications.

Information consulted	Total	Percentage (%)
Dose	172	29%
General information	165	28%
Use and dose	92	15%
Adverse reaction	41	7%
Interactions	36	6%
Pharmaceutical alternative	30	5%
Administrative	25	4%
Adverse reactions + Use	14	2%
Contraindications	10	2%
Storage	5	1%
Allergies	4	1%
Total	594	100%

Among the drugs consulted there were a total of 226 different active ingredients, and according to their category ATC 2nd level classification there were a total of 61 categories, however, the ATC 2nd level classification which represent 80% of the total, are described in Table 2, among the most representative are: sex hormones and modulators of the genital system, of which the most representative principle was levonorgestrel or popularly known as the “morning after pill”. The second most representative category was that of analgesics and anti-inflammatory and antirheumatic products in which the most prominent active ingredients were diclofenac and acetaminophen. On the other hand, in antihistamines for systemic use and preparations for cough and cold, the most consulted active ingredients were: chlorphenamine, acetylcysteine, dextromethorphan, combination ambroxol and clenbuterol, cetirizine, nasal sodium chloride, phenylephrine, and montelukast; the last most representative group was that of vaccines in which the main one was that of human papillomavirus with 30% of the total vaccines consulted.

**Table 2:** Main medications consulted according to category ATC 2nd level classification.

ATC 2nd level classification	Total	Percentage (%)
Sex Hormones and Modulators of the Genital System	57	10%
Analgesics	47	9%
Anti-Inflammatory And Antirheumatic Products	46	8%
Antihistamines For Systemic Use	34	6%
Cough and Cold Preparations	32	6%
Vaccines	27	5%
Agents Against Obstructive Tract Disorders	25	5%
Antibacterial For Systemic Use	25	5%

## 4 Discussion

The analysis of consultations and symptom distribution in the dataset revealed several key findings. The dataset comprised 1537 entries categorized into pharmaceutical recommendations (48%), medication information (31%), other consultations (18%), and referrals to another health professional (3%). Most pharmaceutical recommendations (90%) were classified as minor ailments, predominantly falling into the categories of digestive, respiratory, and moderate pain. Prescription drugs accounted for 75.3% of the recommended and consulted medicines, while over-the-counter (OTC) drugs represented 24.7%. However, considering the proposed BTC category, the percentage of recommended prescription drugs decreased to 29.6%, while BTC drugs constituted 45.7%. Statistical analysis using the chi-square test indicated a significant association between minor ailments and the availability of OTC drugs, rejecting the null hypothesis of independence.

Regarding the most frequently recommended medications, it can be observed from Fig. 2 that individuals between the ages of 18 and 65 were the most prevalent age group seeking these services. This age range represents a considerable portion of the population, which is noteworthy because typically the primary demographic for pharmaceutical recommendations includes patients over 65 years old, who often have multiple comorbidities and require multiple medications.^21^^,^^22^

When considering the mode of sale for the recommended drugs, 75.3% of the medications were classified as prescription drugs in the country. However, this emphasizes the need for the expansion or establishment of a third category, namely BTC, as most of the recommended drugs in this study would fall within this category. These findings are particularly relevant given that, in our country, patients often visit pharmacies first to seek solutions for their symptoms, with pharmacists playing a key role in treating these pathologies.

Considering the decree proposed by health authorities in Costa Rica, a total of 207 different active ingredients were identified, of which over a third are included in the list of medications that can be dispensed without a prescription. This highlights the need for standardization and protocolization of such medications, along with establishing referral criteria to other healthcare professionals. This step could bring about improvements in healthcare, like in Canada and UK where pharmacists have the authority to prescribe medications for specific conditions.^23^ This also underscores the necessity for pharmacists to possess clinical reasoning skills to diagnose minor ailments based on symptoms and make well-informed and technical decisions. Another significant aspect of pharmaceutical practice is that patients can effectively prevent the recurrence of minor ailmentsif pharmacists equip them with the necessary tools. In other words, the education provided by pharmacists plays a vital role in primary prevention, which involves intervening before health effects occur. This can be achieved through measures such as vaccinations, modifying risky behaviors and prohibiting substances and actions known to be linked to specific diseases or health conditions.^23^

As presented in Fig. 2, most medications recommended by pharmacists belong to the genitourinary system and sex hormones group, which can be attributed to the approval of Levonorgestrel (emergency contraception) in Costa Rica in 2019.

Another significant aspect is medications that act on the nervous system, including analgesics and anti-inflammatories. Caution should be taken while prescribing these medications, and patients should be informed about their adverse effects and the risk associated with chronic use, particularly non-steroidal anti-inflammatory drugs, and their gastro-harmful and cardiovascular profile.^24^

Pharmacists commonly prescribe medications for conditions related to the digestive system and metabolism, which is consistent with the high density of consultations related to these issues. Therefore, the implementation of official protocols to treat these pathologies becomes increasingly important. A study conducted in Costa Rica proposed a protocol that focuses on nausea, vomiting, heartburn, constipation, and diarrhea, which may be useful in managing these consultations.^25^

The consultations that involved information about medications showed that patients mostly had doubts about the dosage and general information about the medication. These consultations included several inquiries such as dosage, use, interactions, and contraindications in a single visit to the pharmacy. This suggests that patients are interested in responsible self-medication and seek the advice of a healthcare professional to manage their symptoms. Thus, it is important to regulate this practice with protocols to ensure patient safety.^4^^,^^5^^,^^10^^,^^23^

Table 2 presents the main categories of the ATC classification that patients consulted, with sex hormones again being the top category as previously discussed. The category of analgesics and anti-inflammatories also appears again, along with preparations for coughs and colds. This emphasizes the importance of regulating and standardizing these therapeutic groups, as they are frequently consulted by patients in pharmacies. Additionally, the high frequency of consultations for respiratory symptoms highlights the need for early detection of respiratory pathologies, which may require referral to medical professionals for accurate diagnosis and treatment.

A statistical test was performed to evaluate the dependence between the type of minor ailment for which the patient consulted and whether the indicated medication belongs to the list of active ingredients of OTC medications. The findings from both chi-square tests indicate a meaningful relationship between the availability of OTC drugs and the type of minor ailments experienced by patients. These results hold important implications for healthcare professionals, policymakers, and individuals seeking appropriate medication for their minor ailments. It suggests that the choice of OTC or non-OTC drugs should be guided by the type of symptoms experienced. Tailoring the availability and recommendation of OTC drugs based on the nature of the ailment could potentially enhance treatment outcomes and improve patient satisfaction. Regarding the availability of medications recommended by pharmacists, it is imperative to establish regulations that align with the country's context. The creation of a BTC list should not merely be a customary practice but should also address the actual needs of the population. This process should be approached responsibly, focusing on the treatment of minor ailments while consistently emphasizing the significance of medical consultations.

### 4.1 Limitations

The current study must be recognized for its inherent limitations. Firstly, the study's restricted scope to a sample of pharmacy users poses a challenge in generalizing the findings to the wider population of Costa Rica. Furthermore, a notable limitation arises from the lack of available evidence on pharmaceutical recommendations in other countries, making it difficult to compare the study's results. This can be attributed to the pioneering nature of the topic within the region, emphasizing the need for further research and exploration in this area.

## 5 Conclusions

The analysis of consultations in pharmacies reveals that the majority of cases involve minor ailments, particularly digestive symptoms, joint pain, and respiratory issues. Considering the proposed decree by health authorities in Costa Rica, it is notable that over a third of the identified active ingredients can be dispensed without a prescription. This highlights the necessity for standardization and protocolization of these medications, along with the establishment of referral criteria to other healthcare professionals. Following the examples set by other countries, granting pharmacists the authority to prescribe medications for specific conditions could lead to improvements in healthcare.

## Funding

This research was not funded by any external sources.

## Declaration of Competing Interest

The authors declare that there are no conflicts of interest.

## Appendix A Supplementary data

Supplementary material: List of OTC drugs in Costa Rica. Extracted from the Costa Rican Legal Information System (Nº 35595-S Declaration of Over-the-Counter Drugs to the Consumer) Image 1
