# Supplementary material for: Assessing pharmaceutical consultations: Comparing pharmacy-recommended medications for minor ailments and regulatory compliance in a Latin American healthcare network
Source: Explor Res Clin Soc Pharm. 2023 Jul 6;11:100300. doi: 10.1016/j.rcsop.2023.100300 (PMC10371813; doi:10.1016/j.rcsop.2023.100300)
Supplement: Supplementary file 1 — Supplementary material: List of OTC drugs in Costa Rica. Extracted from the Costa Rican Legal Information System (Nº 35595-S Declaration of Over-the-Counter Drugs to the Consumer) [file mmc1.docx]

**List of OTC drugs in Costa Rica. Extracted from the Costa Rican Legal Information System (Nº 35595-S Declaration of Over-the-Counter Drugs to the Consumer)**

a) Analgesics-Anti-inflammatories: Liquid preparations for oral use must be free of ethyl alcohol.

a. General pain relief:

i. Acetaminophen, up to 750 mg per tablet or capsule, alone or combined with caffeine, and/or with antacids at regulated concentrations in the corresponding section.

ii. Acetylsalicylic acid, up to 500 mg per tablet or capsule, alone or combined with caffeine, and/or antacids at regulated concentrations in the corresponding section.

b. Relief of dysmenorrhea:

i. Ibuprofen 200 mg per tablet or capsule.

ii. Naproxen 220 mg per tablet or capsule. (This subparagraph was added by Executive Decree No. 36041 of May 10, 2010)

c. Relief of mouth pain, teething, mouth ulcers:

i. Benzocaine up to 2% in ointment, gel, or solution.

ii. Chamomile flower extract up to 37% in aerosol.

iii. Lidocaine hydrochloride up to 2%.

iv. Matricaria powder.

d. Topical relief of muscle pain:

i. Diclofenac gel 1%.

ii. Diclofenac sodium 1% in aerosol.

iii. Ibuprofen cream 10%.

iv. Indomethacin gel 1%.

v. Ketoprofen gel 2.5%.

vi. Porous patches.

vii. Belladonna and/or arnica patches.

viii. Preparations with capsaicin in patches, creams, or lotion up to 0.075% capsaicin.

ix. Methyl salicylate with camphor and/or menthol in essential oil-based ointments, creams, lotions, powders, and gels.

b) Antacids and Gastrointestinal Agents:

a. Antacids and Stomach Remedies:

i. Sodium bicarbonate.

ii. Activated charcoal.

iii. Calcium carbonate.

iv. Aluminum carbonate.

v. Magnesium carbonate.

vi. Dimenhydrinate 25 mg and 50 mg tablets.

vii. Pure chamomile flower extract in solution.

viii. Famotidine up to 10 mg per tablet, for adult use only.

ix. Aluminum hydroxide.

x. Magnesium hydroxide.

xi. Magnesium and aluminum hydroxycarbonate, alone or combined with charcoal, simethicone, or dimethylpolysiloxane up to 25 mg per dose.

xii. Effervescent salts with magnesium citrate, magnesium sulfate, and sodium bicarbonate.

xiii. Simethicone or dimethicone up to 150 mg per tablet.

b. Laxatives:

i. Mineral oil.

ii. Ispaghula husk.

iii. Fibers.

iv. Senna leaves.

v. Magnesium magma.

vi. Calcium polycarbophil.

vii. Psyllium.

viii. Effervescent salts with magnesium citrate, magnesium sulfate, and sodium bicarbonate.

c. Antidiarrheals:

i. Lactobacillus.

ii. Oral rehydration salts according to the WHO formula or with lower sodium content

iii. For adult use only:

1. Kaolin + pectin.

2. Loperamide 2 mg in capsules, tablets, or coated tablets.

3. Bismuth subsalicylate.

d. Hypocholesterolemic:

i. Ispaghula husk.

c) Cough, Colds, and Sore Throat: Liquid preparations for oral use must be free from ethyl alcohol.

a. Colds: The following combinations are accepted:

i. Antihistamine + Decongestant + Antitussive.

ii. Antihistamine + Decongestant + Expectorant.

iii. Antihistamine + Decongestant + Expectorant + Analgesic.

iv. Antihistamine + Decongestant + Antitussive + Expectorant.

v. Antihistamine + Decongestant + Antitussive + Expectorant + Analgesic.

vi. Antihistamine + Decongestant + Antitussive + Analgesic.

vii. Antihistamine -+- Antitussive.

viii. Antihistamine -+- Antitussive + Expectorant.

ix. Antihistamine -+- Antitussive + Analgesic.

x. Antihistamine + Expectorant.

xi. Antihistamine + Analgesic.

xii. Decongestant + Antitussive.

xiii. Decongestant + Antitussive + Expectorant.

xiv. Decongestant + Antitussive + Expectorant + Analgesic.

xv. Decongestant + Antitussive + Analgesic.

xvi. Decongestant + Expectorant.

xvii. Decongestant + Expectorant + Analgesic.

Caffeine and/or vitamin C up to 100 mg per tablet or 0.5 mL of solution or drops may be added. The following are accepted in these combinations:

xviii. Analgesics:

1. Acetaminophen up to 750 mg per tablet or capsule or 100 mg/mL in drops or 150 mg/5 mL.

2. Acetylsalicylic acid up to 500 mg per tablet or capsule.

xix. Antihistamines:

1. Chlorpheniramine maleate up to 4 mg per tablet or capsule, or 0.5 mg/mL in drops, or 2 mg/5 mL in syrup.

2. Brompheniramine maleate up to 4 mg per tablet or capsule, or dexbrompheniramine maleate up to 0.3 mg/mL in syrup.

xx. Decongestants: Liquid preparations must be free from ethyl alcohol.

1. Phenylephrine bitartrate or hydrochloride up to 10 mg per tablet or capsule or 5 mL in syrup.

2. Pseudoephedrine HCl up to 60 mg per tablet or capsule or 6 mg/mL in syrup.

b. Antitussives and Expectorants:

i. Camphor 12.5%, rosemary essence 5%, eucalyptus essential oil 5%, Peru balsam 6% in ointment.

ii. Dextromethorphan HBr up to 30 mg per tablet or capsule or up to 0.2% in syrup.

iii. Thyme extract, sundew, drosera 8%, thyme oil 0.19% in drops.

iv. Guaifenesin up to 100 mg per tablet or 100 mg/5 mL in syrup.

v. Guaiacol up to 0.2% in syrup.

Liquid preparations must be free from ethyl alcohol. They can be combined with each other or with cold formulas or accepted bronchodilators such as ephedrine sulfate or hydrochloride up to 0.3%.

c. Sore throat:

i. Tablets containing cetylpyridinium up to 2.5 mg, benzocaine up to 10 mg, or oxybuprocaíne hydrochloride 0.2 mg, alone or in combination, based on menthol, eucalyptol, and other natural essences.

d) Otic products:

a. Benzocaine up to 2% with antipyrine up to 5% in drops.

e) Ophthalmic products:

a. Antiseptics:

i. Boric acid up to 0.03%.

b. Vasoconstrictors:

i. Naphazoline up to 0.01%.

ii. Tetrahydrozoline up to 0.05%.

c. Lubricants:

i. Hydroxypropyl methylcellulose up to 2.5%.

ii. Sodium carboxymethylcellulose up to 5 mg/mL.

f) Foot Care:

a. Athlete's Foot:

i. See section on Topical Antifungals.

b. Callus Removers:

i. Salicylic acid up to 40%.

g) Hemorrhoids and Circulatory System:

a. Hemorrhoid Products:

i. Preparations in suppositories, ointments, or creams containing the following active ingredients: 1% yeast cell extract, 3% shark liver oil with or without 5% lidocaine.

ii. 5% tribenoside, 2% lidocaine cream.

b. Circulatory System:

i. Dry extract of Horse Chestnut 100 mg per tablet + Concentrated Mirotol 10 mg + Rutin 300 mg.

h) Hair and Scalp:

a. Antiparasitics:

i. 25% benzyl benzoate lotion.

ii. Pyrethrins up to 3% alone or with piperonyl butoxide.

i) Hay Fever and Allergies: Liquid preparations for oral use must be free of ethyl alcohol.

a. 0.04% chlorpheniramine maleate syrup.

b. 4 mg and 8 mg chlorpheniramine maleate tablets.

c. 10 mg loratadine tablets.

d. Other formulas used for colds (see “Colds” in section C) of this executive decree).

e. Nasal decongestants and/or emollients with 0.9% sodium chloride, 0.25% and 0.5% phenylephrine, or 0.025% and 0.05% oxymetazoline, and 0.05% and 0.1% xylometazoline.

j) Children:

a. Pain and Teething:

i. See “Relief of mouth pain, teething, mouth ulcers” in section a of this executive decree.

ii. Acetaminophen 100 mg/mL in drops or 150 mg/5 mL.

b. Gastrointestinal:

i. See “Antacids and Stomach Remedies” and “Laxatives” in section aof this executive decree.

c. Colds and Allergies:

i. See “Colds” and “Antitussives and Expectorants” in section c and section i of this executive decree.

d. Cough:

i. See “Antitussives and Expectorants” in section c and section i of this executive decree.

e. Skin and Scalp:

i. See “Antiparasitics” in section h of this executive decree.

f. Diaper Rash:

i. Creams based on cold cream.

ii. 1% w/w dry extract of chamomile flowers cream.

iii. Ointments or creams containing zinc oxide, cod liver oil as active ingredients.

g. Tonics and Other Dietary Supplements:

i. See section o of this executive decree.

h. Sleep and Travel Products, Antiemetics:

i. Dimenhydrinate 25 mg per tablet.

ii. See section m of this executive decree.

i. Stomatitis:

i. Gentian violet solution up to 2%.

k) Oral Care:

a. Mouthwashes and tablets:

i. See “Sore Throat” in section c of this executive decree.

ii. Mouthwashes for breath.

iii. Drops and tablets for breath.

iv. Hydrogen peroxide 3% (hydrogen peroxide).

b. Products for Tooth Decay and Plaque:

i. Toothpaste and mouthwashes with fluorides, with or without antiseptics.

c. Sensitive Teeth:

i. Desensitizing toothpaste containing 5% potassium nitrate.

l) Antiseptics and Skin Care Products:

a. Antiseptics:

i. Aluminum acetate, powder.

ii. Hydrogen peroxide (topical solution).

iii. Mercurochrome.

iv. Thimerosal.

v. Gentian violet.

vi. Iodine.

vii. Povidone-iodine solution 10%.

b. Acne Products in creams, lotions, gels, and soaps:

i. Benzoic acid up to 5% + Salicylic acid up to 10%, sulfur up to 10%, ichthammol up to 3%.

ii. Salicylic acid up to 10%, sulfur up to 10%, and lanolin.

iii. Sulfur up to 10%, triclosan up to 4%, calamine.

iv. Benzoyl peroxide up to 10%.

c. Skin Irritation and Itching:

i. Sublimed sulfur up to 10%, citronella oil, methyl salicylate up to 0.5%.

ii. Benzocaine up to 5% or lidocaine up to 5%.

iii. Calamine with or without antiallergic and local anesthetic.

iv. Crotamiton 10% in lotion and cream.

v. 2% w/w chamomile flower extract cream.

vi. Lubricants: Mineral oil, diethyltoluamide jelly, and essential oils.

d. Protectors and Emollients:

i. Insect repellents based on diethyltoluamide and essential oils.

e. Topical Antifungals:

i. Undecylenic acid with Triclosan up to 1%.

ii. Bifonazole up to 1%.

iii. Clotrimazole up to 1% in cream for topical and vaginal use.

iv. Ketoconazole cream up to 2%.

v. Miconazole topical cream or powder up to 2%, vaginal cream up to 2%, and vaginal tablets up to 100 mg for 3-day treatment.

vi. Terbinafine up to 1%.

vii. Tolnaftate up to 1%.

f. Topical Antibiotics:

i. Bacitracin up to 0.5%.

ii. Bacitracin up to 0.5% with Neomycin up to 0.5%.

iii. Neomycin up to 0.5%.

iv. Mupirocin up to 2%.

v. Oxytetracycline hydrochloride up to 3% w/w with Polymyxin B.

m) Sleep-inducing Adjunct Products: Liquid preparations for oral use must be free from ethyl alcohol.

a. Restlessness and Insomnia:

i. St. John's Wort extract 250 mg tablets.

ii. Valerian extract, tablets or 8% syrup.

iii. Valerian extract 250 mg -+- Hops extract 60 mg coated tablets.

iv. Valerian root 65 mg/tablet + Passiflora incarnata up to 65 mg -+- hops powder 32.5 mg per tablet, with or without other extracts with similar effects.

b. Motion Sickness Adjunct:

i. Dimenhydrinate 25 mg and 50 mg tablets.

n) Stimulants and Tonics: Liquid preparations for oral use must be free from ethyl alcohol.

a. B-complex Vitamins, Glycerophosphates, Soft Extracts, Caffeine.

o) Vitamins, Minerals, and Nutrients: Liquid preparations for oral use must be free from alcohol.

a. Dietary Supplements:

i. Soy lecithin up to 1200 mg per capsule.

ii. Nutritional supplements based on proteins, carbohydrates, fats, vitamins, and minerals up to 50% of the Recommended Daily Allowance (RDA) per dosage unit, whether dairy-based or not, intended for consumption by individuals without special nutritional or therapeutic requirements.

iii. Vitamin C alone up to 500 mg per tablet or 500 mg per 5 mL of syrup or 100 mg per mL in drops.

iv. Single vitamins or multivitamins with or without minerals that do not exceed 150% of the accepted daily requirement (RDA).

p) Wrappers or envelopes with:

a. Sulfur.

b. Sodium bicarbonate (baking soda).

c. Magnesium citrate.

d. Crémor tartar.

e. Sulfur flowers.

f. Linden flowers.

g. Senna leaves.

h. Lycopodium.

i. Zinc oxide.

j. Simple talc.

q) Auxiliary products for smoking cessation:

a. Nicotine up to 14 mg/patch.

b. Nicotine 2% in chewable tablets.

**BTC drug proposal. Extracted from the draft of the Pharmaceutical Indication Commission's Proposal. (Not a public or official document, the list is being drafted and managed in coordination with the Costa Rican Ministry of Health, the College of Pharmacists, and the commissions involved in the process.)**

The following medications are declared as pharmaceutical indication medications and therefore can only be offered in establishments that have a Sanitary Operating Permit for Pharmacy and with the appropriate advice from the pharmaceutical manager:

6.1 Medications for fever

• Sodium/potassium diclofenac

• Dexketoprofen

• Nimesulide

6.2 Medications for cough

• Ambroxol

• Carbocisteine

• Cloperastine

• Dropropizine

• Hedera helix

• N-acetylcysteine

• Levodropropizine

• Levocloperastine

• Oxolamine

6.3 Medications for nasal congestion

• Cetirizine

• Cetirizine/Pseudoephedrine

• Chlorpheniramine

• Diphenhydramine

• Desloratadine

• Fexofenadine

• Levocetirizine

• Rupatadine

6.4 Medications for insomnia

• Diphenhydramine

• Doxylamine

• Hydroxyzine

6.5 Medications for anxiety

• Magnesium glutamate

• Sulpiride

6.6 Contraceptive medications

• Dihydroxyprogesterone acetophenide/Estradiol enantate

• Cyproterone acetate/Ethinylestradiol

• Clormadinone acetate/Ethinylestradiol

• Nomegestrol acetate/17Beta-Estradiol

• Desogestrel

• Desogestrel/Ethinylestradiol

• Dienogest/Ethinylestradiol

• Dienogest/Valerate estradiol

• Drospirenone/Ethinylestradiol

• Norethisterone enantate/Valerate estradiol

• Etonogestrel

• Etonogestrel/Ethinylestradiol

• Gestodene/Ethinylestradiol

• Linestrenol

• Levonorgestrel

• Levonorgestrel/Ethinylestradiol

• Medroxyprogesterone

• Norgestrel/Ethinylestradiol

6.7 Medications for vaginal fungal infections

• Butoconazole

• Clindamycin

• Fluconazole

• Itraconazole

• Itraconazole/Secnidazole

• Ketoconazole

• Metronidazole

• Metronidazole/Miconazole

• Metronidazole/Nystatin

• Nystatin

• Secnidazole

• Sertaconazole

• Terbinafine

• Tinidazole

• Tinidazole/Fluconazole

6.8 Medications for back pain

• Celecoxib

• Dexibuprofen

• Dexketoprofen

• Sodium/potassium diclofenac

• Sodium/Piridoxine/Thiamine/Cyanocobalamin/Hydroxocobalamin diclofenac

• Sodium/Piridoxine/Thiamine/Cyanocobalamin diclofenac

• Etoricoxib

• Ketoprofen

• Meloxicam

• Piroxicam

• Sulindac

6.9 Medications for headache

• Dexibuprofen

• Dexketoprofen

• Sodium/potassium diclofenac

• Ketorolac

• Piroxicam

6.10 Migraine Medications

• Acetaminophen / Caffeine / Dihydroergotamine

• Acetaminophen / Caffeine / Atropine Belladonna / Ergotamine

• Caffeine / Propyphenazone / Meclozine / Camphor / Ergotamine

• Clonixin / Ergotamine

• Desibuprofen

• Dexketoprofen

• Sodium/potassium diclofenac

• Ketorolac

• Piroxicam

6.11 Dental Pain Medications

• Dexketoprofen

• Sodium/potassium diclofenac

• Etoricoxib

• Ibuprofen

• Lysine

6.12 Sore Throat Medications

• Ambroxol

• Bencidamine

• Benzalkonium chloride / Lidocaine

• Benzocaine

• Cetylpyridinium

• Cetylpyridinium / Hydrocortisone / Benzocaine

• Chlorhexidine gluconate

• Decylaminium / Betaglycyrrhetinic acid / Hydrocortisone / Lidocaine

• Decylammonium chloride / Dibucaine

• Dexketoprofen

• Sodium/potassium diclofenac

• Lysine

• Nimesulide

6.13 Menstrual Pain Medications

• Mefenamic acid

• Desketoprofen

• Lysine

• Sodium metamizole (Dipyrone)

6.14 Heartburn Medications

• Esomeprazole

• Famotidine

• Lansoprazole

• Levosulpiride

• Metoclopramide

• Pantoprazole

• Magnesium pantoprazole

• Sucralfate

6.15 Diarrhea Medications

• Bacillus Clausii

• Lactobacillus reuteri / Glutamine

• Lactobacillus casei, Lactobacillus rhamnosus, Streptococcus thermophilus, Bifidobacterium, Lactobacillus acidophilus, Bifidobacterium longum, Lactobacillus bulgaricus

• Nifuroxazide

• Saccharomyces boulardii

6.16 Constipation Medications

• Bisacodyl

• Glycerin

• Lactulose

• Methylcellulose

• Picosulfate

• Polyethylene glycol

6.17 Hemorrhoid Medications

• Diosmin

• Dobesilate / Lidocaine

• Dobesilate / Lidocaine / Dexamethasone

• Protocain / Cinchocaine

• Polycresulen

• Polycresulen / Cinchocaine

• Tribenoside

• Tribenoside / Lidocaine

6.18 Nausea Medications

• Doxylamine / Pyridoxine

• Meclizine

• Metoclopramide

6.19 Vomiting Medications

• Meclizine

6.20 Dyspepsia Medications

• Amylase / Lipase / Protease

• Big Man extract

• Butylscopolamine

• Butylscopolamine / Metamizole

• Lactase

• Levosulpiride

• Metoclopramide

• Otilonium bromide

• Pancreatin

• Pancreatin / Simethicone

• Pancreatin / Simethicone / Bromopride

• Propinoxate

• Propinoxate / Lysine clonixinate

• Simethicone

6.21 Medications for Irritable Bowel Syndrome

• Amylase / Lipase / Protease

• Scopolamine

• Lactase

• “Big Man” extract

• Butylscopolamine

• Butylscopolamine / Metamizole

• Otilonium bromide

• Pancreatin

• Pancreatin / Simethicone

• Pancreatin / Simethicone / Bromopride

• Propinoxate

• Propinoxate / Lysine clonixinate

6.22 Acne Medications

• Fusidic acid

• Salicylic acid / Benzoic acid / Sulfur / Ichthyol

• Clindamycin

6.23 Diaper Rash Medications

• Silicone oil

• Silicone oil / Silver

• Acexamic acid

• Acexamic acid / Neomycin

• Dexpanthenol

• Levomenol / Matricaria Chamomilla / Achillea Millefolium

• Zinc oxide / Dexpanthenol / Vitamin E / Vitamin A

• Zinc oxide / Vitamin A / Vitamin D

• Dexpanthenol

• Dexpanthenol / Aloe Barbadensis

• Dexpanthenol / Retinol / Vitamin E / Zinc

• Dexpanthenol / Glycerin / Copper, Magnesium, and Zinc

• Dexpanthenol / Hyaluronic acid / Copper, Magnesium, and Zinc

• Retinol / Calcium, Magnesium, and Zinc

• Retinol / Colecalciferol / Vitamin E / Matricaria Chamomilla

• Silver sulfadiazine / Lidocaine / Retinol

• Vitamin E

6.24 Cold Sore Medications

• Acyclovir

• Valacyclovir

6.25 Urticaria Medications

• Betamethasone

• Cetirizine

• Diphenhydramine

• Desloratadine

• Fexofenadine

• Hydrocortisone

• Levocetirizine

• Loratadine / Betamethasone

• Rupatadine

6.26 Conjunctivitis Medications

• Fusidic acid

• Carbomer / Cetrimide

• Chondroitin / Hyaluronate

• Hyaluronate

• Hydroxypropylmethylcellulose / Dextran

• Polyethylene glycol / Propylene glycol

• Povidone

• Propylene glycol

• Tobramycin

6.27 Dry Eye Medications (Lubricants)

• Carbomer / Cetrimide

• Chondroitin / Hyaluronate

• Hyaluronate

• Hydroxypropylmethylcellulose

• Hydroxypropylmethylcellulose / Dextran

• Polyethylene glycol / Propylene glycol

• Povidone

• Propylene glycol

6.28 Ear Infection Medications

• Antipyrine / Benzocaine / Glycerin

• Benzocaine / Benzalkonium / Glycerin

• Carbamide peroxide

• Glycerin

• Tetracaine / Dexamethasone

6.29 Skin Infection Medications

• Fusidic acid

• Mupirocin

• Colloidal silver

• Sulfadiazine

• Sulfadiazine / Lidocaine / Retinol

6.30 Dermatophytosis Medications

• Benzoic acid / Boric acid / Salicylic acid

• Salicylic acid / Triclosan / Aluminum and Potassium

• Bifonazole / Urea

• Ciclopirox

• Clotrimazole / Hydrocortisone

• Ketoconazole / Salicylic acid / Undecylenic acid

• Miconazole

• Terbinafine

• Tolnaftate

6.31 Wound Healing Medications

• Silicone oil

• Silicone oil / Silver

• Acexamic acid

• Clobetasol / Neomycin

• Dexpanthenol

• Lanolin

• Zinc oxide / Dexpanthenol / Vitamin E / Vitamin A

• Zinc oxide / Vitamin A / Vitamin D

• Dexpanthenol

• Dexpanthenol / Aloe Barbadensis

• Dexpanthenol / Retinol / Vitamin E / Zinc

• Dexpanthenol / Glycerin / Copper, Magnesium, and Zinc

• Dexpanthenol / Hyaluronic acid / Copper, Magnesium, and Zinc

• Retinol / Calcium, Magnesium, and Zinc

• Retinol / Colecalciferol / Vitamin E / Matricaria Chamomilla

• Silver sulfadiazine / Lidocaine / Retinol

• Vitamin E

6.32 Varicose Veins Medications

• Diosmin / Hesperidin

• Calcium dobesilate

• Hidrosmin

6.33 Immunostimulants

• Escherichia coli

• Hedera helix / Pelargonium sidoides

• Lyophilized Bacterial Lysate of Haemophilus Influenzae 7 mg; Streptococcus (Diplococcus) pneumoniae, Klebsiella pneumoniae ssp. pneumoniae and ozaenae; Staphylococcus aureus; Streptococcus pyogenes and sanguinis (viridans); Moraxella (Branhamella/Neisseria) catarrhalis.

• Pelargonium sidoides

• Pidotimod

6.34 Vaccines

• Bacillus Calmette-Guérin (BCG)

• Diphtheria / Pertussis / Pertussis / Haemophilus influenzae type B / Inactivated Poliovirus (DTaP-Hib-IPV)

• Diphtheria / Pertussis / Pertussis / Haemophilus influenzae type B / Inactivated Poliovirus / Hepatitis B (DTaP-Hib-IPV-HepB)

• Yellow Fever

• Haemophilus influenzae type B (Hib)

• Hepatitis A (HepA)

• Hepatitis B (HepB)

• Hepatitis A and B (HepA-HepB)

• Recombinant Quadrivalent Influenza (RIV)

• Meningococcal A, C, W, Y (MenACWY)

• Meningococcal B (MenB)

• Conjugate Pneumococcal (PCV13)

• Polysaccharide Pneumococcal (PCV23)

• Inactivated Poliovirus (IPV)

• Rotavirus (RV)

• Measles / Mumps / Rubella (MMR)

• Tetanus / Diphtheria / Pertussis (Tdap, DTaP)

• Tetanus / Diphtheria Toxoid (Td)

• Varicella (VAR)

• Human Papillomavirus (HPV)

In case the pharmacy professional identifies situations that require medical diagnosis or the expertise of another healthcare professional, they should refer the patient, considering the following factors:

7.1 If the symptoms have persisted for a considerable period.

7.2 If the condition has recurred or worsened.

7.3 If there is acute pain.

7.4 If one or more medications that seemed appropriate for treating the symptoms have been tried without obtaining positive results.

7.5 If there are suspected adverse reactions to prescription or over-the-counter medications.

7.6 If the symptoms have been recognized as severe.
